# Understanding cancer from the perspective of cellular adaptation to stressors for survival

**DOI:** 10.1002/qub2.70048

**Published:** 2026-08-02

**Authors:** Zhenyu Huang, Xuechen Mu, Ying Xu

**Affiliations:** ^1^ Systems Biology Lab for Metabolic Reprogramming Department of Human Genetics and Cell Biology School of Medicine Southern University of Science and Technology Shenzhen China

**Keywords:** fenton reaction, genomic mutations, intracellular alkalosis, metabolic reprogramming, stress adaptation

## 1 INTRODUCTION

In microbial and plant studies, organismic evolution is generally believed to be driven by persistent stressors, internal or external, and adaptation for survival [1]. Specifically, stressors lead to increased oxidative stress and, hence, an increased mutation rate, and some of the mutations are selected to start metabolic reprogramming to adapt to or mitigate the stressors. For example, *Escherichia coli* under persistent ethanol stress could lead to reduction of its ATP production due to membrane leakage from oxidative damages by ethanol; this would result in reduced biosynthesis of large biomolecules, such as phospholipids, forming a vicious cycle that gives rise to progressively decreased ATP generation and ultimately death [2]. Some cells adapt to the stressor by generating and selecting loss‐of‐function mutations in genes encoding major ATP‐consuming proteins and utilize the saved ATP toward the biosynthesis of phospholipids, which are then used to repair the damaged membrane, leading to an increased level of ATP production and forming a positive cycle toward full recovery of ATP production and cellular functions [3]. Similarly, *Arabidopsis thaliana* cells adapt to drought stress through the creation and selection of a combination of three mutations [4], becoming drought tolerant.

If the same evolutionary principle, namely, “stressors → increased mutations or epigenomic changes → some are selected to start certain reprogrammed metabolisms for adaptation,” applies to human cancer, a natural question is as follows: What stressors may drive cancer formation and progression across possibly all cancer types because all cancers, at least all solid cancers, share substantial commonalities such as the hallmarks of cancer [5] and highly conserved reprogrammed metabolisms such as de novo nucleotide biosynthesis and sialic acid biosynthesis [6]? The following addresses this question.

## 2 HOW DO HUMAN CELLS SENSE, COMMUNICATE, AND RESPOND TO A LIFE‐THREATENING STRESSOR?

The essence of severe stress is that changes in certain aspects of the microenvironment, extracellular or intracellular, affect the cell’s health and function, and the cell’s capabilities encoded in its genome, in the form of a large set of **IF (X) THEN (Y)** commands, are inadequate to cope with the stress, which may become progressively damaging, leading to cell death if not dealt with.

During their life cycle, human cells may experience a variety of stressors, such as those imposed by microenvironmental changes including temperature, pH, redox balance, hypoxia, and DNA damage. These stressors converge on endoplasmic reticulum (ER) stress. When the ER stress reaches a certain level that threatens the cell’s health or even its livelihood, the cell has several ways in its toolbox to deal with the stress, namely, (1) systematically seeking **Y**, while ignoring condition **X** as in the above **IF‐THEN** command, that can help adapt to or mitigate the stress through epigenetic regulation; (2) expanding the available toolbox through dedifferentiation or transdifferentiation to increase the chance of finding tools that can help adapt to the stress; or (3) generating new survival pathways through creating and selecting genetic mutations.

Computational detection of such relationships could be conducted through statistical analyses of transcriptomic and genomic data, such as correlation analysis, regression analysis, and deep learning‐based causal inference. For example, information about cellular states and the levels of cellular functions conducted by biological processes could be inferred based on the expression of the relevant marker genes. Relationships among the cellular states and biological processes could be derived through correlation and regression analyses coupled with chemical reaction equations, further validated through causal inference [7].

## 3 WHAT STRESSORS MAY DRIVE CANCER ONSET AND PROGRESSION?

Our previous studies [6, 8, 9, 10] have discovered that all cancers in The Cancer Genome Atlas (TCGA) harbor Fenton reactions, respectively, in the extracellular matrix, cytosol, and mitochondria of epithelial cells or similar cell types that are easy to divide:

(1) $${\text{Fe}}^{2+}+H_{2}O_{2}\;\to \;{\text{Fe}}^{3+}+{\text{OH}}^{-}+\cdot \text{OH}$$

as a result of chronic inflammation, namely, persistently elevated concentrations of $H_{2}O_{2}$ and $\cdot O_{2}$ released by innate immune cells such as neutrophils and macrophages, and local accumulation of ${\text{Fe}}^{2+}$. If there are reducing molecules nearby, ${\text{Fe}}^{3+}$ can be reduced back to ${\text{Fe}}^{2+}$, driving the reaction to continue as long as there are plenty of such reducing molecules. In the cancer and cancer‐forming microenvironment, we have demonstrated that $\cdot O_{2}$ serves the reducing role to convert ${\text{Fe}}^{3+}$ to ${\text{Fe}}^{2+}$. Hence, the persistent Fenton reactions can be written in the following manner:

(2) $$H_{2}O_{2}+\cdot O_{2}\;\overset{{\text{Fe}}^{2+}}{\to }\;{\text{OH}}^{-}+\cdot \text{OH}+O_{2}.$$

It is noteworthy that persistent Fenton reactions are the result of the oxidative molecule ($H_{2}O_{2}$) meeting the reductive molecule ($\cdot O_{2}$) in the presence of ${\text{Fe}}^{2+}$. Our cell‐based experiment has demonstrated that vitamin C can serve the same role as $\cdot O_{2}$ in driving persistent Fenton reactions (Supporting Information S1: Figure S1).

During the inflammatory and precancer stages, the affected tissues generally have Fenton reactions in the extracellular matrix first. The persistently produced hydroxyl radical ·OH, as the most reactive free radical that human cells can produce, causes damage to both the extracellular matrix and the associated epithelial cells, hence driving progressively more immune cells, initially innate immune cells, and gradually more adaptive immune cells, such as T cells, to the sites, all for tissue repair and, hence, chronic inflammation [11]. Note that cancer has long been considered a wound that will not heal. As the disease progresses, persistent Fenton reactions start to take place in the cytosol (and mitochondria), which continuously produce ${\text{OH}}^{-}$ and drive up the intracellular pH. This information was derived through analyses of the transcriptomic data of each tissue sample along with detection and application of the relevant marker genes [6, 8].

Under such persistent alkalization, the intracellular pH buffer and $H^{+}$ pumps are not adequate to keep the pH stable due to their limited capacity and the restraint that cells’ electroneutrality cannot be violated [10]. The affected cells start acidifying, namely, $H^{+}$‐producing metabolisms, induced via the above stress response process(es), to keep the intracellular pH stable, as changes in this pH would affect the overall chemistry in the cells and, hence, cells viability [10].

We have also studied the transcriptomic data of all noncancerous diseases, consisting of both cancer‐prone and cancer‐independent diseases in the Gene Expression Omnibus (GEO) database, and found that all these diseases each harbor intracellular Fenton reactions. A major discovery is that the cancer‐prone and cancer‐independent diseases can be well distinguished based on how the levels of intracellular Fenton reactions change as a disease evolves. Specifically, all cancer‐prone diseases each harbor intracellular Fenton reactions that progressively increase, whereas each cancer‐independent disease has intracellular Fenton reaction(s) that plateau at a relatively low level compared with those in cancer‐prone diseases (Figure 1A,B; manuscript in preparation).

**FIGURE 1:**
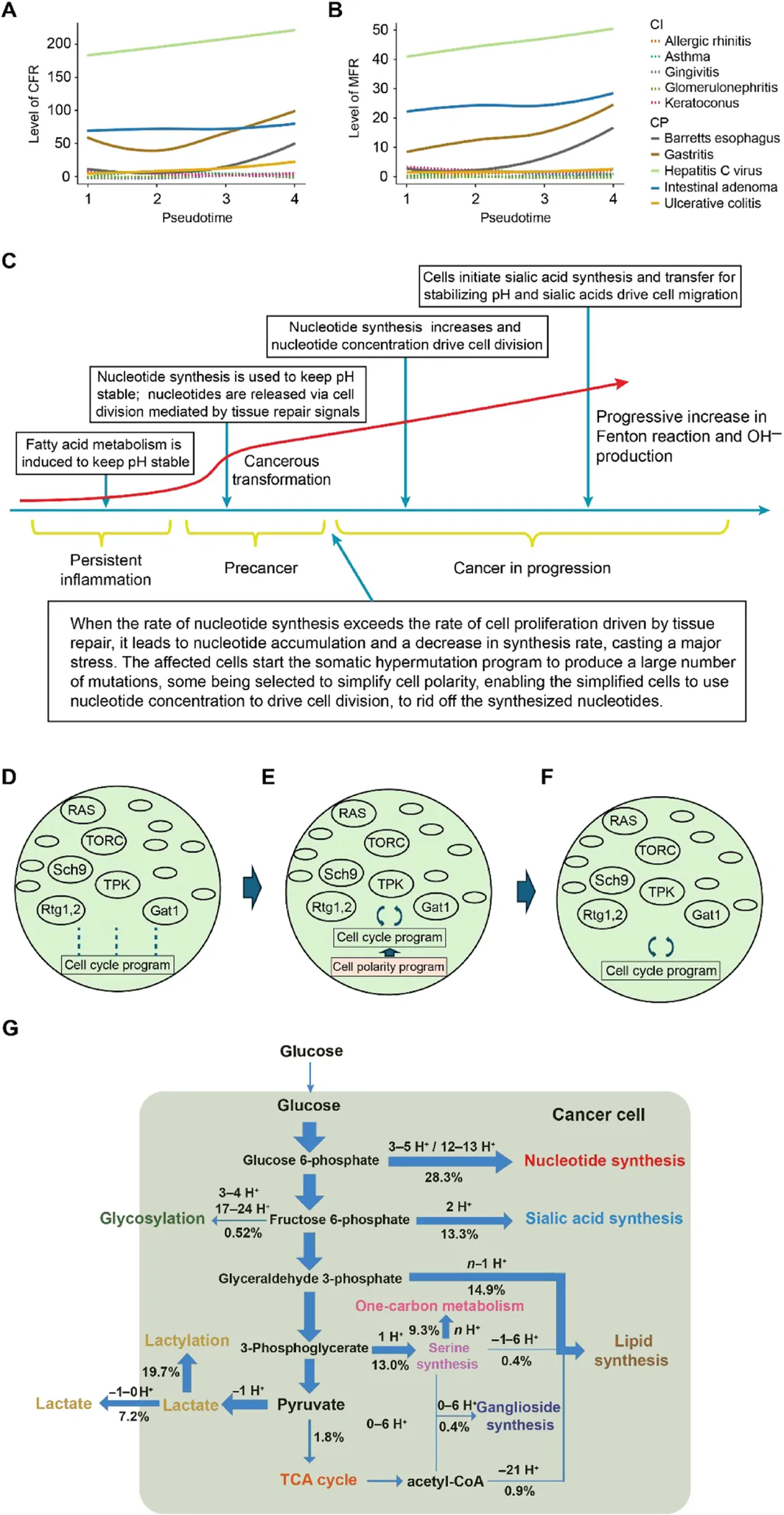
Stress‐driven cancer formation and the evolutionary basis of cancer cell‐cycle programs. (A) Levels of cytosolic Fenton reaction (CFR) in cancer‐prone (CP) and cancer‐independent (CI) diseases. Five CP diseases (Barrett’s esophagus, gastritis, hepatitis C virus infection, intestinal adenoma, and ulcerative colitis) and five CI diseases (allergic rhinitis, asthma, gingivitis, glomerulonephritis, and keratoconus) are analyzed. Transcriptomic data are retrieved from the GEO database. For each disease, samples are ordered in ascending order of their transcriptomic “distance” of selected enzyme genes from the average of matching normal tissues in the GTEx database (manuscript in preparation). The *x*‐axis represents the ranked samples (referred to as the pseudotime of disease progression, normalized to 1%–100%), and the *y*‐axis represents the level of CFR. (B) Levels of MFR across the same set of diseases, with axes defined as in (A). (C) A schematic of the key evolutionary stages in cancer formation and progression, where the *x*‐axis is for disease progression and the *y*‐axis is for the level of CFR. (D) Nutrient concentration‐driven cell‐cycle program in yeast, where sensors, each represented as an oval, for nutrients communicate the levels of nutrients of various types and combinations to signaling and growth pathways, leading to the apparatus of cell division. (E) The human cell‐cycle program has evolved from one in unicellular organisms, which has added a layer of polarity genes (the red part), which receives signals from the development or tissue‐repair program for cell division and then sets the infrastructure for cell division, which is more complex than that in unicellular organisms, and starting the cell‐cycle program for nutrient preparation. (F) Cancerous cells have largely mutated the cell‐polarity functions added throughout multicellular organism evolution from unicellular organisms, essentially converting the human cell‐cycle program to one similar to that in yeast. (G) Metabolic flux analyses of glucose in cancer cells, where the numbers above and below each arrow represent the number of protons produced per glucose and the percentage of glucose uptake through the reaction represented by the arrow, respectively. GEO, Gene Expression Omnibus; GTEx, Genotype‐Tissue Expression; MFR, mitochondrial Fenton reaction; TCA, tricarboxylic acid cycle.

Another discovery we made is that cancer in TCGA generally evolves in a manner as depicted in Figure 1C. During the inflammatory stage, the level of cytosolic Fenton reaction (CFR) is at a relatively low level, where simple acidifying metabolisms, such as fatty acid metabolism and post‐translational modifications such as phosphorylation and glycosylation, are deployed and are adequate to keep the intracellular pH stable. As the disease advances, the Fenton reaction level increases, making these metabolisms inadequate to maintain a stable pH. With the progressively increased alkaline stress, more powerful acidifying metabolisms, such as de novo (deoxy)nucleotide synthesis, are induced to keep the pH stable, where the synthesis of a purine also produces 8–9 $H^{+}$ ions and a pyrimidine 3–5 $H^{+}$ ions [6]. To make this process sustainable, cells need to get rid of the synthesized nucleotides in a timely manner.

It should be noted that it is not simple to release the nucleotides extracellularly because they each carry a negative charge, and their persistent release will violate the cell’s electroneutrality and, hence, is prohibited. As a result, the synthesized nucleotides are released through synthesizing them into nascent DNA, mediated by cell division for tissue repair (Note: The other side of inflammation is tissue repair), and then by having the electroneutral DNA‐histone complexes placed into the new daughter cell, completing the process of releasing the nucleotides. This process, termed the precancerous stage in Figure 1C, continues so long as the rate of cell division is fast enough for releasing the synthesized nucleotides in a timely manner, namely, without their intracellular accumulation.

As the CFR level continues to rise, the level of nucleotide synthesis must increase to keep up with the level of ${\text{OH}}^{-}$ production by Fenton reactions. The affected cells would be under considerable stress when the nucleotide synthesis rate becomes higher than the cell‐division rate, resulting in intracellular accumulation of the synthesized nucleotides and, hence, slowing down nucleotide synthesis and $H^{+}$ production, giving rise to progressively increased alkaline stress.

When the stress level reaches a certain threshold, the somatic hypermutation program, normally used only by B cells for antibody production, is activated via dedifferentiation and epigenomic regulation to produce large numbers of mutations (manuscript in preparation), different from what the literature has suggested, namely, mutations being the result of oxidative stress or DNA replication errors [12]. Among the selected mutations, a large fraction is in genes that are homologous to yeast genes for nutrient sensing or signaling such information to the cell‐cycle program; we term this group Type 1. Another large proportion is in genes of the cell‐polarity programs [13], namely, those used to build the infrastructure in support of the accurate and efficient execution of all the biological functions needed for cell division; we designate this group Type 2. Our analyses have revealed that genes in Type 1 are all proto‐oncogenes and overexpressed and genes in Type 2 are predominantly tumor suppressor genes (manuscript in submission). This discovery provides biologically more meaningful definitions for these widely used terms.

Further analyses have revealed that genes in Type 1 tend to have copy‐number increases across different cancer types, and the cell‐polarity genes, after excluding genes in Type 2, are mostly homologous to yeast cell‐polarity genes. Overall, the selected point mutations considerably simplify the cell‐polarity program to one like that in yeast, where nucleotide concentration can drive cell division [14]. As a result, the synthesized nucleotides can be released extracellularly in a timely manner through cell division, as shown in Figure 1C.

Overall, when cells are affected by intracellular Fenton reactions, resulting from persistent inflammation and local iron accumulation, they will be persistently alkalized, killing the cells by alkalosis if not neutralized. Under such stress, cells utilize the somatic hypermutation program to create and select large numbers of genomic mutations to simplify the cellular infrastructure for cell division so that nutrient concentrations can drive the cell‐cycle program, as in yeast, leading to cell division for the purpose of releasing the synthesized nucleotides in a timely manner, as depicted in Figure 1D–F. It is important to note that Fenton reaction‐affected cells may give rise to other chronic diseases such as Alzheimer’s disease [15]—it is the nature of the underlying cell types that dictates which acidifying metabolic reprogramming might be activated and the disease types. For epithelial or similar cell types that are easy to divide, nucleotide synthesis is deployed to produce $H^{+}$ and keep the intracellular pH stable, and cell division provides a natural metabolic exit for the synthesized nucleotides. For neural cells, cell division is very difficult, and hence the affected cells utilize other acidifying metabolisms to keep the pH stable, for example, hydrolysis of glutamine to glutamate plus $H^{+}$, leading to hyperexcited neurons when the glutamate is released and ultimately massive neuronal death. Similar patterns are also observed in other diseases, such as idiopathic pulmonary fibrosis (in preparation), abdominal aortic aneurysm (in preparation), and Type 2 diabetes (in preparation), namely, intracellular Fenton reactions drive acidifying metabolic reprogramming to keep the pH stable, whose types are dictated by the organ and cell types, giving rise to different disease phenotypes.

From this perspective, it is largely the level of the intracellular Fenton reaction that dictates the rate of cancerous cell division. Based on this, we state that cancer is a disease where cells use cell division as a way for survival; hence, stopping cell division will kill the cancer cells.

## 4 WHY DO CANCER CELLS REQUIRE A LARGE AMOUNT OF GLUCOSE?

It has been well established that cancer cells take up considerably more glucose than the corresponding normal cells, which has been the basis for cancer detection using positron emission tomography‐computed tomography (PET/CT). The traditional explanation for this is that cancers grow fast and, therefore, need a lot of glucose as nutrients. However, the reality is different. Flux analyses of glucose metabolism across multiple cancer types show, as shown in Figure 1G, that, on average, glucose in cancer tissue cells mainly flows into de novo nucleotide synthesis (28.3%), lipid synthesis (14.9%), sialic acid synthesis (13.3%), serine synthesis (13.0%), and a few minor acidifiers. These metabolic fluxes account for more than 70.0% of the consumed glucose on average, predominantly for $H^{+}$ production plus slightly more than 0.5 ATP (= [19.7 + 7.2]% × 2 ATP) per glucose. In other words, a majority of the taken‐up glucose is used to generate $H^{+}$ to maintain cytoplasmic pH stability. Without this glucose, cells would die from alkalosis, suggesting that inhibiting glucose from entering cancer cells might be an effective way to kill cancer cells.

Although the total rate of glucose consumption by a cancer tissue largely reflects the level of its CFR, its flux distribution and the levels across different glucose metabolic branches can inform about the characteristics of a cancer. For example, the higher the flux toward nucleotide synthesis, the faster the cancer grows [6]. Similarly, the faster the sialic acids accumulate, measured using sialic acid synthesis and sialic acid degradation, the more malignant the cancer is [16]. Supporting Information S1: Figures S2 and S3 show the fluxes of glucose metabolism across different stages of highly versus mildly malignant cancer (sub)types, along with information on how the flux data provide characteristic information about a cancer (sub)type, such as the malignancy level (Supplementary Information, “Two‐step flux balance analysis”).

For normal human cells, glucose is a nutrient that can be used to produce ATP or converted to the basic building blocks for synthesizing macromolecules, such as amino acids and fatty acids. Without glucose, cells can survive on fats and amino acids, but cancer cells will die from alkalosis, as has been demonstrated by other authors [17] and our own study (Supporting Information S1: Figure S4; manuscript in preparation).

## 5 WHAT IS THE RELATIONSHIP BETWEEN THE FENTON REACTION AND FERROPTOSIS IN NORMAL AND CANCER CELLS?

Ferroptosis is caused by the interaction between hydroxyl radicals, produced by Fenton reactions, and phospholipids, generating lipid fragments that are toxic to the host cell and leading to cell death. For this to happen, Fenton reactions must occur near the cell membrane, as hydroxyl radicals travel, on average, no more than 1 nm. In cancers, CFR primarily occur in the labile iron pool, distant from the cell membrane; therefore, they generally do not cause ferroptosis.

## 6 SIMILARITIES AND DIFFERENCES IN STRESSORS IN PRIMARY CANCER AND METASTATIC CANCER CELLS AND THEIR MECHANISMS IN COPING WITH THESE STRESSORS

Metastasized cancers have been treated like primary cancers based on the premise that they behave somewhat similarly and have basically the same genomes. A key difference between them is that primary cancers alone generally do not kill the patients, whereas metastasized cancers are responsible for more than 93% of cancer‐associated deaths. It has been a puzzling issue of why medicine designed for treating primary cancer generally does not work on metastasized cancers. The reality is more complex than it seems on the surface.

Unlike primary cancer cells, whose main and driving stressor is intracellular alkalization, metastatic cancer cells face primarily hyperoxic stress based on our comparative analyses of transcriptomic data of the four types of cancers, namely, metastasized cancers in bone, brain, liver, and lung. Primary cancer cells have hypoxic microenvironments. As a result of evolution, such cells lost their key barrier that prevents $O_{2}$ from entering the cell, which can oxidize key biomolecules and kill the cell as a result. It has been established that it is the cholesterol content in the cell plasma membrane that serves as that barrier to keep $O_{2}$ out [18]. Our analyses of transcriptomic data of such tissues have confirmed that primary cancer cells gradually lose a major portion of their plasma membrane cholesterol content (manuscript in preparation). Therefore, when they migrate to a blood‐rich and, hence, $O_{2}$‐rich microenvironment, they experience significant hyperoxic stress. As the main stress response, the metastasized cells continuously uptake and synthesize large amounts of cholesterol to replenish the depleted cholesterol in the cell plasma membrane, which is also under attack by the complement system in the metastasized sites, damaging their cell plasma membranes. Under high hyperoxic conditions, most cholesterol entering the cells becomes oxidized and, therefore, cannot be used to repair the cell membrane; instead, it is further metabolized to sex hormones and glucocorticoids, where the former are powerful growth signals and the latter are T‐cell inhibitors, which together give rise to the unique behavioral characteristics of metastatic cancers, namely, almost uninhibited explosive growth (manuscript in preparation).

## 7 CONCLUSION

In sum, it is the persistent alkaline stressors that drive cancer onset and development through the induction of acidifying programmed metabolisms to keep the intracellular pH stable for cellular survival, where genomic mutations are created and selected to enable the induction of the reprogrammed metabolisms as stress responses, based on our analyses of transcriptomic data of cancer tissues, coupled with computational chemistry and computational biology analyses. Our study has revealed that metastasized cancers are fundamentally different from their primary counterparts as they are driven by different stressors and adapted by the host cells via different mechanisms compared to primary cancers; therefore, metastasized cancers should be treated differently.

## AUTHOR CONTRIBUTIONS

**Zhenyu Huang**: Conceptualization; data curation; formal analysis; investigation; validation; visualization; writing—original draft. **Xuechen Mu**: Conceptualization; data curation; formal analysis; investigation; validation; visualization; writing—original draft. **Ying Xu**: Conceptualization; funding acquisition; investigation; methodology; project administration; supervision; writing—original draft; writing—review and editing.

## CONFLICT OF INTEREST STATEMENT

Ying Xu is the Editorial Board Member of *Quantitative Biology*. He was excluded from the peer‐review process and all editorial decisions related to the acceptance and publication of this article. Peer review was handled independently by the other editors to minimize bias. The other authors declare no conflicts of interest.

## ETHICS STATEMENT

This study used only publicly available, de‐identified data and involved no new experiments on humans or animals. Accordingly, institutional review board approval and informed consent were not required.

## Supporting information

Supporting Information S1

## Data Availability

All datasets analyzed in this study are publicly available from the following repositories: TCGA via the GDC portal (portal.gdc.cancer.gov), GTEx (gtexportal.org), NCBI GEO (ncbi.nlm.nih.gov/geo), KEGG (kegg.jp), and BRENDA (brenda‐enzymes.org).
