# Supplementary material for: Understanding cancer from the perspective of cellular adaptation to stressors for survival
Source: Quant Biol. 2026 Aug 2;14(3):e70048. doi: 10.1002/qub2.70048 (PMC13642068; doi:10.1002/qub2.70048)
Supplement: Supplementary file 1 [file QUB2-14-e70048-s001.docx]

# **SUPPLEMENTARY INFORMATION**

1. **Two-step flux balance analysis**

To estimate glucose-derived flux allocation across eight metabolic pathways in cancer tissues, we developed a two-step flux balance analysis (FBA) [1] pipeline. In the first step, transcriptomic data were integrated with stoichiometric constraints under a pseudo-steady-state assumption to infer cancer-specific catalytic rate constants $\text{k}_{\text{cat}}$, which subsequently informed flux capacity constraints. In the second step, flux allocation was estimated as a quadratic programming (QP) problem that combines mass-balance constraints, activity-derived capacity bounds, and a quadratic regularization term to produce biologically plausible flux distributions.

1.1 **Metabolic network and internal nodes**

We constructed a glucose-centric stoichiometric model based on KEGG pathway data [2]. The network includes four major modules:

a. *Glucose uptake:* Glucose transporters SLC2A1, SLC2A3, SLC5A1, SLC5A2, and SLC2A10 mediate glucose entry and conversion to glucose-6-phosphate (G6P).
b. *Pentose phosphate and glycolysis:* G6P is directed either to the pentose phosphate pathway for nucleotide synthesis or to fructose-6-phosphate (F6P). F6P is further routed to UDP-GlcNAc synthesis, supporting sialic acid production and glycosylation, or proceeds through glycolysis to glyceraldehyde-3-phosphate (G3P).
c. *Lipid synthesis and serine metabolism:* G3P contributes to triglyceride synthesis or is converted to 3-phosphoglycerate (3P), which is further metabolized to serine or pyruvate. Serine can remain as free serine, enter one-carbon metabolism, or support ceramide synthesis, which subsequently feeds sphingolipid and ganglioside synthesis.
d. *Pyruvate metabolism:* Pyruvate is converted either to lactate or to acetyl-CoA. Acetyl-CoA then forms citrate, supporting the tricarboxylic acid (TCA) cycle and lipid synthesis.

In this network, G6P, F6P, UDP-GlcNAc, G3P, 3P, serine, ceramide, pyruvate, acetyl-CoA, and citrate are modeled as internal transit metabolites. Terminal outputs, including nucleotide synthesis, sialic acid, glycosylation products, lipids, lactate, TCA intermediates, gangliosides, one-carbon products, and residual serine, are modeled as sink nodes.

1.2 **Estimation and validation of cancer-specific** $\text{k}_{\text{cat}}$ **values**

a. *Stoichiometric network reconstruction:* KEGG KGML files were parsed for each pathway to obtain reaction identifiers, balanced stoichiometries, enzyme commission (EC) numbers, and gene-protein-reaction (GPR) mappings, yielding a stoichiometric matrix ($\text{S}$).
b. *Enzyme activity proxy matrix (*$\text{E}$*):* Significantly up-regulated enzymes were identified by comparing cancer and normal expression profiles using independent two-sample t-tests followed by Benjamini-Hochberg false-discovery-rate correction [3]. Enzymes with an adjusted p-value < 0.05 and fold-change ≥ 1.5 were retained. This fold-change threshold was used only for $\text{k}_{\text{cat}}$ estimation and was not applied to downstream differential-expression filtering for flux estimation. For reaction $\text{j}$ in cancer sample $\text{i}$, the enzyme activity proxy $\text{E}_{\text{ij}}$ was defined as:

$$\text{E}_{\text{ij}}\text{=}\left\{ \begin{aligned} \max_{\text{g}\text{∈}\text{R}_{\text{j}}}\left( \text{expression}_{\text{i}}\left( \text{g} \right) \right)\text{, if any }\text{g}\text{∈}\text{R}_{\text{j}}\text{ is upregulated} \\ \text{0, otherwise} \end{aligned} \right.$$

(1)

where $\text{R}_{\text{j}}$ denotes the gene set associated with reaction $\text{j}$.

c. *Non-negative least squares optimization (NNLS) for* $\text{k}_{\text{cat}}$ *estimation:* Flux through reaction $\text{j}$ in sample $\text{i}$ was modeled as:

$$\text{v}_{\text{ij}}\text{=}\text{k}_{\text{cat}}\text{×}\text{E}_{\text{ij}}$$

(2)

Under the pseudo-steady-state assumptions, mass balance is expressed as:

$$\text{S}\text{∙}\text{v}\text{=0}$$

(3)

Unknown $\text{k}_{\text{cat}}$ values were estimated by solving a regularized non-negative least squares (NNLS) problem:

$$\min_{\text{k}_{\text{cat}}\text{≥0}}\text{∥}\text{S}\left( \text{k}_{\text{cat}}\text{∘}\text{E} \right)\text{∥}_{\text{2}}^{\text{2}}\text{+}\text{γ}\text{∥}\text{k}_{\text{cat}}\text{-}\text{k}_{\text{cat}}^{\left( \text{0} \right)}\text{∥}_{\text{2}}^{\text{2}}\text{,}$$

(4)

where $\text{k}_{\text{cat}}^{\left( \text{0} \right)}$ denotes initial estimates derived from BRENDA literature priors [4] and CatPred predictions [5], scaled by reaction-specific mean enzyme-gene expression. The regularization parameter $\text{γ}$ balances stoichiometric consistency with prior biological knowledge.

The matrix $\text{E}$ was used only as a design matrix for learning $\text{k}_{\text{cat}}$ and should not be interpreted as a hard activity indicator or direct flux bound. When $\text{E}_{\text{ij}}\text{=0}$ for a reaction without tumor-upregulated enzymes in sample $\text{i}$, that sample does not contribute to the data-fit term for reaction $\text{j}$ in Eq. (5), but the corresponding pathway flux is not forced to zero. The parameter $\text{k}_{\text{cat}\text{,}\text{j}}$ remains informed by literature and machine-learning priors, other samples with $\text{E}_{\text{ij}}\text{>0}$, and NNLS regularization. Downstream flux estimation instead uses DE-filtered ssGSEA–$\text{k}_{\text{cat}}$ capacity bounds.

1.3 **Flux estimation via quadratic programming**

Using the estimated $\text{k}_{\text{cat}}$ values, we modeled glucose-derived flux allocation into eight metabolic pathways as a QP problem. This formulation enforces mass balance while discouraging biologically unrealistic winner-take-all allocation patterns.

1.3.1 **Variables and constraints**

a. *Glucose uptake (*$\text{v}_{\text{glc}\text{\_}\text{in}}$*)*: Total glucose influx mediated by the specified glucose transporters.
b. *Mass-balance constraints*: For each internal metabolite $\text{m}$:

$$\sum_{\text{r}\text{∈influx(}\text{m}\text{)}} \text{v}_{\text{r}}\text{-}\sum_{\text{r}\text{∈efflux}\left( \text{m} \right)} \text{v}_{\text{r}}\text{=0}$$

(5)

c. *Sink reactions*: Terminal pathways, such as nucleotide synthesis, lactate production, TCA intermediates, and ganglioside synthesis, were represented by sink variables ($\text{v}_{\text{sink}}$) that consume glucose-derived carbon exiting the internal nodes.
d. *Flux balance constraint*: Total sink flux equals total glucose uptake:

$$\sum_{\text{sink}} \text{v}_{\text{sink}}\text{=}\text{v}_{\text{glc\_in}}$$

(6)

Minor metabolic sinks, such as hyaluronic acid synthesis, were assumed to be negligible.

e. *Flux capacity constraints*: For each sink reaction, an upper bound ($\text{B}_{\text{sink}}$) was defined using a combination of the pathway activity score $\text{G}_{\text{sink}}$ (computed via ssGSEA, with differentially expressed genes identified first via Welch’s t-test and Benjamini–Hochberg correction; the resulting mean normalized enrichment score was clamped to non-negative values and divided by the square root of the gene-set size for normalization) and the pathway-level normalized catalytic rate coefficient ($\text{k}_{\text{cat}}$, derived from the estimation in Section 5.2 and normalized such that the coefficients across all pathways sum to one): For each sink reaction, the upper bound ($\text{B}_{\text{sink}}$) was defined as:

$$\text{B}_{\text{sink}}\text{=}\text{G}_{\text{sink}}\text{×}\text{k}_{\text{cat}}$$

(7)

Here, $\text{G}_{\text{sink}}$ is the DE-filtered ssGSEA pathway activity score. Differentially expressed genes within each pathway were first identified using Welch's t-test with Benjamini-Hochberg correction; ssGSEA was then applied to the DE genes, and the resulting mean normalized enrichment score was clamped to non-negative values and divided by the square root of the gene-set size. The pathway-level $\text{k}_{\text{cat}}$ coefficient was normalized so that all pathway coefficients summed to one.

Thus, each flux variable satisfies:

$$\text{0≤}\text{v}_{\text{sink}}\text{≤}\text{B}_{\text{sink}}$$

(8)

1.3.2 **Objective function**

To avoid unstable or overly concentrated flux distributions, we maximized a balanced objective that combines linear pathway weights with a quadratic penalty:

|  | $\max_{\text{v}_{\text{sink}}} \sum_{\text{sink}} \left( \text{w}_{\text{sink}}\text{∙}\text{v}_{\text{sink}}\text{ - }\text{λ}\text{∙}\text{v}_{\text{sink}}^{\text{2}} \right)$ | (9) |
| --- | --- | --- |

The pathway weight $\text{w}_{\text{sink}}$ was obtained by applying square-root dampening to $\text{B}_{\text{sink}}$ followed by sum-to-one normalization:

$$\text{w}_{\text{sink}}\text{=}\frac{\sqrt{\text{B}_{\text{sink}}}}{\sum_{\text{sink}^{\text{'}}} \sqrt{\text{B}_{\text{sink}^{\text{'}}}}}\text{ .}$$

(10)

The quadratic term $\text{λ}\text{∙}\text{v}_{\text{sink}}^{\text{2}}$ penalizes disproportionately large sink fluxes, thereby promoting balanced solutions.

Overall, the QP framework integrates stoichiometric mass balance, catalytic-capacity constraints, and expression-derived pathway activity scores to estimate biologically grounded glucose-flux distributions.

2. **Net sialic-acid accumulation**

Metabolic fluxes provide useful information regarding the characteristics of a cancer or cancer type. For example, we computed a mean-expression-based net sialic-acid accumulation level for each sample, where sialic-acid synthesis was estimated using the glucose-uptake-scaled absolute flux through the sialic-acid branch, whereas the degradation rate was approximated by the mean expression of the canonical sialic-acid degradation genes *NEU1, NEU2, NEU3, NEU4*, and *NPL*. Considering that flux and gene expression are measured at different scales, both quantities were standardized across samples before subtraction, yielding a unitless index defined as $\text{z}_{\text{flux}}-\text{z}_{\text{degradation}}$.

This accumulation score was visualized across PAAD (pancreatic cancer), STAD-diffuse (diffuse subtype of stomach cancer), CHOL (bile duct cancer), THCA (thyroid cancer), PRAD prostate cancer), and STAD-intestinal (intestinal subtype of stomach cancer) in Figure S4, where {PAAD, CHOL, STAD-diffuse} are known to be more malignant than {STAD-intestinal, THCA, PRAD}.

The resulting distribution shows that malignant tumors (PAAD, STAD-diffuse, CHOL) display higher levels of sialic-acid accumulations than the mild cancer (sub)types (THCA, PRAD, STAD-intestinal). At the (sub)type level, the median score was positive in the malignant tumors (0.9769) while negative in the mild cancer (sub)types (−0.1780).

# **SUPPLEMENTARY FIGURES AND LEGENDS**

**Figure S1.** Vitamin C drives persistent Fenton reaction and cell proliferation in a cell-based experiment. Duration of cytosolic Fenton reactions (x-axis) and the associated proliferation level (y-axis), along with different levels and combination of the inducing agents in human liver cancer cells HCCLM3 and Huh7. **A-B,** cells treated with different combinations of conditions. **C-D,** Cell treated with different levels of FeSO₄ alone, relative to untreated control (0 μM). **E-F,** Cell treated with different levels of H_2_O_2_. NC, negative control. * *P* < 0.05, ** *P* < 0.01, *** *P* < 0.001, **** *P* < 0.0001.

**Figure S2**. The percentage of glucose import allocated to eight metabolic pathways across cancer stages in multiple cancer types. **A**–**F** show glucose import flux allocation in CHOL, PAAD, PRAD, STAD-diffuse, STAD-intestinal, and THCA, respectively. Abbreviations: CHOL, cholangiocarcinoma; PAAD, pancreatic adenocarcinoma; PRAD, prostate adenocarcinoma; STAD, stomach adenocarcinoma; THCA, thyroid carcinoma.

**Figure S3.** Mean-expression-adjusted net sialic-acid fluxes across six tumor subtypes. The net sialic-acid index was calculated for each sample as the standardized glucose-uptake-scaled sialic-acid absolute flux minus the standardized mean expression of sialic-acid degradation enzymes (*NEU1, NEU2, NEU3, NEU4,* and *NPL*).

**Figure S4**. Differential viability and gene expression in cancer and normal cell lines under glucose restriction following Fenton reaction induced alkaline stress. **A**. Live/dead fluorescence staining of gastric and liver cell lines under normal- and low-glucose conditions. **B**. Quantification of cell death rates under normal- and low-glucose conditions. **C.** Relative expression levels of the de novo nucleotide synthesis-related genes TYSM, RRM2, and NEM1 in gastric cell lines GES-1, AGS, and HGC27 cultured under normal-glucose and low-glucose conditions. **D.** Relative expression levels of the de novo nucleotide synthesis-related genes TYSM, RRM2, and NEM1 in liver cell lines THLE2, LM3, and Huh7 cultured under normal-glucose and low-glucose conditions. Abbreviations: GES-1, human gastric epithelial cell line GES-1; AGS, human gastric adenocarcinoma cell line AGS; HGC-27, human gastric carcinoma cell line HGC-27; THLE-2, transformed human liver epithelial-2 cell line; HCCLM3/LM3, human hepatocellular carcinoma lung metastasis cell line HCCLM3; Huh-7, human hepatocellular carcinoma cell line Huh-7; N, normal-glucose condition; L, low-glucose condition; PI, propidium iodide; TYSM, thymidylate synthase; RRM2, ribonucleotide reductase regulatory subunit M2; NEM1, nucleotide metabolism-related enzyme 1. Data are presented as mean ± SD. Statistical significance is indicated as follows: * *P* < 0.05, ** *P* < 0.01, *** *P* < 0.001, **** *P* < 0.0001.

**REFERENCE**

[1]	J. D. Orth, I. Thiele, and B. Ø. Palsson, “What is flux balance analysis?,” *Nat. Biotechnol.*, vol. 28, no. 3, pp. 245–248, Mar. 2010, doi: 10.1038/nbt.1614.

[2]	M. Kanehisa and S. Goto, “KEGG: kyoto encyclopedia of genes and genomes,” *Nucleic Acids Res.*, vol. 28, no. 1, pp. 27–30, Jan. 2000, doi: 10.1093/nar/28.1.27.

[3]	Y. Benjamini and Y. Hochberg, “Controlling the False Discovery Rate: A Practical and Powerful Approach to Multiple Testing”, Accessed: May 03, 2026. [Online]. Available:dx.doi.org/10.1111/j.2517-6161.1995.tb02031.x

[4]	A. Chang, M. Scheer, A. Grote, I. Schomburg, and D. Schomburg, “BRENDA, AMENDA and FRENDA the enzyme information system: new content and tools in 2009,” *Nucleic Acids Res.*, vol. 37, no. Database issue, pp. D588–D592, Jan. 2009, doi: 10.1093/nar/gkn820.

[5]	V. S. Boorla and C. D. Maranas, “CatPred: a comprehensive framework for deep learning in vitro enzyme kinetic parameters,” *Nat. Commun.*, vol. 16, no. 1, p. 2072, Feb. 2025, doi: 10.1038/s41467-025-57215-9.
